# Willingness to pay for an mRNA-based anti-cancer treatment: results from a contingent valuation study in Israel

**DOI:** 10.1186/s13584-024-00594-z

**Published:** 2024-02-19

**Authors:** Omer Ben-Aharon, Ruslan Sergienko, Georgi Iskrov, Dan Greenberg

**Affiliations:** 1 Department of Health Policy and Management, School of Public Health, Faculty of Health Sciences, Ben-Gurion University of the Negev, Beersheba, Israel; 2 Department of Social Medicine and Public Health, Faculty of Public Health, Medical University of Plovdiv, Plovdiv, Bulgaria

**Keywords:** Cancer, Oncology, Willingness to pay (WTP), Contingent valuation, mRNA

## Abstract

**Background:**

mRNA technology is currently being investigated for a range of oncology indications. We assessed the willingness to pay (WTP) of the general population in Israel for a hypothetical novel mRNA-based treatment for oncology indications.

**Methods:**

We used a contingent valuation methodology to elicit WTP using a web-based questionnaire. A sample of adult participants were presented with a hypothetical scenario in which an mRNA-based intervention increased the likelihood of a cure for various cancer types from 20% to 40% (half of the sample), or 60% (the other half of the sample).

**Results:**

531 respondents completed the questionnaire. The mean, median and mode WTP for the proposed hypothetical treatment in both scenarios were ILS65,000 (± ILS114,000), ILS20,000 and ILS50,000, respectively (1USD = 3.4ILS). The WTP was skewed towards zero, and 9.6% of the respondents were not willing to pay any amount. WTP higher amounts was significantly associated with higher income (*p* < 0.01), self-reported good health (*p* < 0.05), supplementary health insurance (*p* < 0.05), Jews compared to other populations (*p* < 0.01), interest in technology (*p* < 0.001) and a tendency to adopt medical innovations (*p* < 0.001). No statistical difference between the 40% vs. the 60% potential cure scenarios was found. Logistic and OLS regressions indicated that age, religion, income, and interest in adopting medical innovations were the best predictors of respondents' WTP.

**Conclusion:**

Despite the scientific breakthroughs in oncology treatment over the last few decades, many types of cancer are still incurable. Given the expected development of innovative mRNA-based treatments for cancer, these results should inform policymakers, the pharmaceutical industry and other stakeholders on the future coverage and reimbursement of these technologies incorporating patients’ and societal views. To date, WTP considerations have not been given much weight in prioritization of drug reimbursement processes, neither in Israel nor in other countries. As a pioneer in adoption of the mRNA technology, Israel can also lead the incorporation of WTP considerations in this field.

## Introduction

In 2020, messenger-RNA (mRNA)-based vaccines were presented as a promising alternative to conventional vaccine approaches [1]. This prospect was associated with the capacity for rapid development, low manufacturing costs and safe administration. Although the potential of mRNA-based health technology had been described in the media, mRNA was only actually implemented on a broad scale later in 2020, after emergency authorizations by the FDA in the US and the EMA in the EU to manufacture mRNA COVID-19 vaccines. The uptake of this technology was then one of the fastest in the history of medicine, where revenues for mRNA vaccines skyrocketed from almost zero in 2020 to over $50 billion in 2022 [2, 3].

The COVID-19 vaccination campaigns based on mRNA technology demonstrated high levels of safety and efficacy/effectiveness in both clinical trials [4, 5] and real-world settings in many countries [6, 7]. The rapid rollout of COVID-19 vaccinations in Israel was very impressive, and by the end of 2020, the State of Israel, with a population of 9.3 million, had administered more COVID-19 vaccine doses than all other countries aside from China, the US, and the UK [8]. Israel also pioneered a third dose ("booster") campaign prior to official approval by the main regulatory authorities in other jurisdictions [9].

Today, mRNA-based vaccines are being tested for other diseases, including several oncology indications. Some phase 1 and phase 2 studies have begun recruiting patients with locally advanced or metastatic solid tumors, including melanoma, non-small cell lung cancer (NSCLC), bladder cancer, and colorectal cancer. Unlike mRNA-based COVID-19 vaccines that focus on prevention, cancer vaccines are designed to treat tumors by stimulating an immune response [10] and are currently unlikely to be used as stand-alone therapy. Moderna (ModernaTX, Inc.) has two cancer vaccines in clinical trials for the adjuvant treatment of melanoma and solid tumors; in both cases in combination with Merck's Keytruda (Pembrolizumab). The interim results from the phase 2 clinical trials appear promising and have demonstrated a statistically significant and clinically meaningful 44% improvement in the adjuvant treatment of patients with melanoma following complete resection [11]. BioNTech, which partnered with Pfizer in the development of the mRNA COVID-19 vaccine, has several cancer vaccines in development and early-stage clinical trials via FDA fast-track designation for the treatment of melanoma [12].

Now that mRNA-based cancer treatments are likely to be introduced as add-on therapies, these interventions are expected to be subject to value assessment and appraisal to determine coverage and reimbursement policies. Their value needs to reflect patients' and society's preferences with respect to resource allocation and priority-setting. The value of oncology treatments and test information has been examined in many studies [13–15]. Most have implemented a conventional cost-effectiveness/value-for-money framework, which frequently presents the cost per quality-adjusted life year (QALY) gained.

By contrast, assessing individuals’ willingness to pay (WTP) for an intervention constitutes a different approach to value assessment. WTP studies can better approximate patient-centeredness by enabling the scientific inquiry to estimate individual preferences in monetary terms to determine how people value healthcare interventions [16]. The premise is that the benefits derived from goods or services are related to what people are willing to pay for them [17]. Although both the QALYs and the WTP methods are based on individual preferences, their underlying assumptions diverge and yield systematically different conclusions as to the relative value perceived by individuals for health interventions [18]. Due to these differences in normative perceptions, the linear translation from QALYs to WTP is theoretically unattainable [19]. Some studies have even suggested that WTP outperforms QALYs as a measure of the benefits of health care programs [20].

The WTP for a hypothetical vaccine has been examined in several recent studies, including vaccine for the COVID-19 in the U.S. [21], Japan [22] and Chile [23] prior to the launch of mRNA vaccines, as well as vaccine for malaria in Brazil [24].

WTP studies examining oncology interventions worldwide have reported a wide range of valuations which were shown to depend on the intervention, the healthcare system of the country in question, the type of respondents, and other factors affecting the implied monetary value [25]. To the best of our knowledge, none of these studies has assessed the WTP for more advanced technologies in cancer care, such as immunotherapy or mRNA-based oncology interventions. The current study assessed WTP for a novel hypothetical mRNA-based treatment targeted at specific oncology tumors: melanoma, lung cancer, breast/ovarian cancer or cancer of the digestive system. We focused on relatively common oncology indications currently investigated in clinical trials examining mRNA-based treatment.

We hypothesized that WTP for hypothetical innovative treatment would be linked to treatment efficacy, as well as respondents’ demographic characteristics, income and history of illness as found in previous studies.

## Methods

### Participants

A representative sample of the general adult population of Israel was recruited through a web-based panel by the survey firm Midgam Panel. Minimal incentives (ILS 0.5–2.0) for participation in the study were offered to each respondent. The socio-demographic characteristics of the respondents were extracted from the Midgam Panel database. The survey was conducted in May 2022. Each participant was asked a series of questions on a structured questionnaire (see Appendix 1). Information was collected on self-reported health, supplementary and private health insurance coverage, and history of cancer (specifically for this study) as well as other personal characteristics, including age, gender, income, education, and marital status. The remaining questions dealt with respondents’ health risk tolerance and attitude towards technology and medical innovations.

### WTP elicitation

The two most widely used survey-based approaches for eliciting WTP are the contingent valuation method (CVM) and the discrete-choice experiment (DCE). Using DCEs, individuals are asked to select their preferred (and/or least preferred) alternative from a defined set of alternatives [26]. The CVM is a standard economic measure of WTP for health interventions and offers researchers the flexibility to investigate how people value a wide range of health benefits in monetary terms [27, 28]. In general, contingent valuation tasks can be structured to ask respondents about the value they assign to health or the value they assign to a healthcare program [29]. The CVM has been applied to a broad range of different technologies and diseases [30]. Over the past few decades, a considerable number of WTP studies have been conducted using the CVM, thus confirming its validity and reliability [31]. Hence, we adopted in this study the CVM approach for elicitation of WTP.

We examined the WTP of the public in Israel for a hypothetical combination of immunotherapy medication and mRNA-based treatment for melanoma, lung cancer, breast/ovarian cancer or cancer of the digestive system. The CVM was applied to elicit WTP.

### Definition of efficacy of the mRNA intervention

Five-year survival is often considered synonymous in the oncology community for “cure”. According to the American Cancer Society, the 5-year survival rates for the metastatic state of melanoma and NSCLC using current treatment standards of care are 30% [32] and 8% [33], respectively. The hypothetical mRNA treatment presented to participants in this study indicated an increase in the probability of a “cure” from 20% (efficacy of current immuno-oncology agents) to 40% or 60% (for half of the total respondents in each sub-group). The hypothetical scenario presented to the respondents is displayed in Box 1.

**Box 1:** Hypothetical scenario presented to the respondents

Briefly, mRNA technology is one of the outcomes of research on the human genome. Prior to the COVID-19 pandemic, mRNA had little practical medical use. At the end of 2020, the leading health authorities in the U.S. (FDA) and Europe (EMA) approved the commercialization of Pfizer’s and Moderna’s Covid-19 vaccination based on this technology. These are the vaccines used in Israel.	
Now imagine the following theoretical scenario: you contact your general practitioner with complaints about pain and fatigue. After a series of tests, the GP tells you that you have advanced melanoma (skin cancer), lung cancer, breast/ovarian cancer or cancer of the digestive system. In terms of treatment options, the physician suggests either immunotherapy (medication that boosts the body's natural immunization system to fight the tumor), or a combination of immunotherapy and mRNA technology. The first, which is fully reimbursed by your health fund, has a 20% chance of 5-year survival. The addition of mRNA treatment, which is not covered by the basic health insurance package, has been approved by health regulatory authorities in the US (FDA) and in Europe (EMA). It is expected to lead from the current 20% survival rate to a 40% chance of 5-year survival (for half of the respondents)/60% (for the other half of respondents).	
Both treatments are administered once a month in the hospital and have a similar adverse events profile. If you are interested in the second option, you will have to pay for it out of your own pocket regardless of your health plan or complementary private insurance you may have.	
Would you agree to pay XXX (*) to cover the annual expense for f new treatment, which is not covered by your health insurance?	
1) Yes	
2) No, but I would be willing to pay a lesser amount	
3) No, I would not be willing to pay any amount	

(*) XXX –50 respondents in each group (~ 250 respondents per group of 40%/60% probability) were administered the questionnaire with the following amounts NIS20,000; NIS50,000; NIS100,000; NIS250,000; NIS500,000 (overall 10 sub-groups) (1USD = 3.4ILS in May 2022).

### Formulation of the willingness to pay question

To elicit respondents’ WTP, we administered a set of double-bounded dichotomous questions, followed by two open-ended questions. Each participant was presented with a hypothetical scenario describing the new mRNA intervention and an initial bid. They were asked to state whether they would agree to pay the annual expenses of the mRNA treatment which were not covered by their health insurance. To address starting point bias (i.e., the influence of the first bid presented), participants were randomized to one of five initial bids selected ranging from ILS20,000, ILS50,000, ILS100,000, ILS250,000 to ILS500,000. Next, in a follow-up question, the respondents were asked to indicate the maximum price they would pay for the specified benefit (open-ended question).

### Statistical analysis

The characteristics of the respondents are displayed in absolute terms and as a proportion of the entire sample in Table 1. The mean, median, mode and range of WTP values were calculated and are presented in monetary terms (ILS). To examine the association between respondents’ characteristics and WTP for the proposed mRNA treatment, the responses to each questionnaire item were aggregated into up to three categories (except for age, which is a continuous variable). The association with the amount of WTP—a continuous variable—was examined using Pearson correlations for age and independent-samples Kruskal–Wallis or Mann–Whitney U tests for other characteristics. The association with WTP—a dichotomous variable—was examined using a Mann–Whitney U Test for independent samples for age, and a Pearson Chi-square for other characteristics.

**Table 1:** Population characteristics

Characteristic	Variable	Description	Sample respondents, # (%)	
Demographics	Sex	Male	257	(48%)
		Female	274	(52%)
	Age	18–24	82	(15%)
		25–34	125	(24%)
		35–44	104	(20%)
		45–54	88	(17%)
		55–64	70	(13%)
		65 +	62	(12%)
	Family status			
		Single	164	(31%)
		Married	304	(57%)
		Separated/Divorced	54	(10%)
		Widowed	9	(2%)
	Children	0	179	(34%)
		1	59	(11%)
		2	102	(19%)
		3	102	(19%)
		4+	79	(15%)
		Not stated	10	(2%)
	Religion	Jew	431	(81%)
		Christian	21	(4%)
		Muslim	53	(10%)
		Druse	26	(5%)
	Religious identification	Secular	257	(48%)
		Traditional	171	(32%)
		Religious	69	(13%)
		Orthodox	34	(6%)
	Region	Jerusalem	53	(10%)
		North	90	(17%)
		Haifa	86	(16%)
		Center	113	(21%)
		Tel Aviv	85	(16%)
		South	77	(15%)
		West bank	27	(5%)
	Income	No income	33	(6%)
		Far below average	152	(29%)
		Below average	129	(24%)
		Average	119	(22%)
		Above average	53	(10%)
		Far above average	27	(5%)
		Prefer not to state	18	(3%)
	Education	Up to 12 years	188	(35%)
		Non-academic	103	(19%)
		Academic	225	(42%)
		PhD	15	(3%)
Health	Health status	Unknown	1	(0%)
		Excellent	113	(21%)
		Very good	194	(37%)
		Good	170	(32%)
		Not very good	45	(8%)
		Poor	8	(2%)
	Health fund basic only	Yes	112	(21%)
		No	419	(79%)
	Cancer occurrence	Myself	14	(2.6%)
		Relatives or friends	229	(43.1%)
		Both of the above	8	(1.5%)
		None	280	(53%)
	Cancer type	Melanoma/skin	17	(3%)
		Lungs	27	(5%)
		Breast/ovarian	55	(10%)
		The digestive system	31	(6%)
		Some of the above	30	(6%)
		Other tumor	70	(13%)
		Blood	15	(3%)
		Both of the above	7	(1%)
		No cancer	279	(53%)
	Covid vaccine	1 dose	26	(5%)
		2 doses	99	(19%)
		3/4 doses (including booster)	348	(66%)
		None	58	(11%)
	Heard about mRNA	Yes, highly familiar	68	(13%)
		Yes, vaguely	146	(27%)
		No	317	(60%)
Risk	Healthy, balanced diet	Always	50	(9%)
		Usually	244	(46%)
		Sometimes	173	(33%)
		Seldom	55	(10%)
		Never	9	(2%)
	Exercise	Frequently	71	(13%)
		Usually	154	(29%)
		Sometimes	168	(32%)
		Seldom	108	(20%)
		Never	30	(6%)
	Periodic medical checks	Always	81	(15%)
		Usually	175	(33%)
		Sometimes	171	(32%)
		Seldom	89	(17%)
		Never	15	(3%)
	Smoking	More than daily pack	50	(9%)
		Up to weekly pack	51	(10%)
		Occasionally	41	(8%)
		Seldom	50	(9%)
		Never	339	(64%)
Technology	Technology attitude	Early adoption	104	(20%)
		Technology tendency	189	(36%)
		Mature adoption	185	(35%)
		Late adoption	35	(7%)
		Negative attitude	18	(3%)
	Medical innovation attitude	Early adoption	93	(18%)
		Reimbursed treatments	304	(57%)
		Standard of care only	96	(18%)
		Negative attitude	38	(7%)

To examine the association between respondents' characteristics and WTP for the proposed mRNA treatment, two regressions methods were used. A stepwise method optimized the model that identified the variables contributing the most to the regression equation. The association with WTP was examined using a logistic regression. The association with level of WTP was examined using ordinary least squares (OLS) regression and quantile regression.

The data analyses were performed using SPSS version 26 (SPSS Inc. Chicago, Illinois). A *p* value of < 0.05 was considered statistically significant for all analyses.

## Results

### Sample Characteristics

#### Demographics

Six hundred and seven individuals were contacted to participate in the study, and 531 fully completed the questionnaire (87.5% response rate). The respondents’ main characteristics are presented in Table 1. The mean (± SD) age was 41.6 (± 15.4) and 52% of the respondents were women. Approximately 48% of the respondents reported their religious identification as secular, whereas the rest were traditional (32%), religious (13%) or orthodox (6%). The majority of the respondents (59%) reported below average income or no income at all, 26% of respondents reported having an average income or preferred not to answer, and 15% reported above average income. More than one third of the respondents had up to 12 years of schooling, 19% had higher non-academic education and 45% had an academic education.

#### Health and health insurance

Most respondents (90%) reported good, very good, or excellent health. A fifth of the respondents (21%) only had basic universal health coverage from one of the four Israeli health plans, whereas 79% of respondents had complementary health insurance: 45% had supplementary insurance provided by their health plan, or private health insurance and 34% had more than one form of private health insurance. Almost half of the participants (47%) reported a history of cancer: 2.6% concerning themselves, 43.1% concerning relatives or friends and 1.5% for both.

A large proportion (89%) of the sample stated they had been vaccinated for COVID-19, of whom 5% had received one dose, 19% two doses and 66% three or four doses (including the booster shot). Forty percent of the respondents had heard about mRNA technology, of whom 13% were very familiar and 27% were vaguely familiar of the technology.

#### Risk and technology orientation

When asked about healthy behavior, more than half of the respondents (55%) declared maintaining a healthy lifestyle and generally eating a balanced diet, 42% reported doing physical exercise, 48% reported going for periodic medical checks and 73% did not smoke cigarettes at all or seldom. More than half of the participants (55%) considered themselves early adopters or were interested in new technologies, while 75% reported they were early adopters of medical innovations or had a positive attitude towards reimbursed treatment for innovations.

### Distribution of the maximum WTP for the hypothetical mRNA treatment

**Fig. 1:**
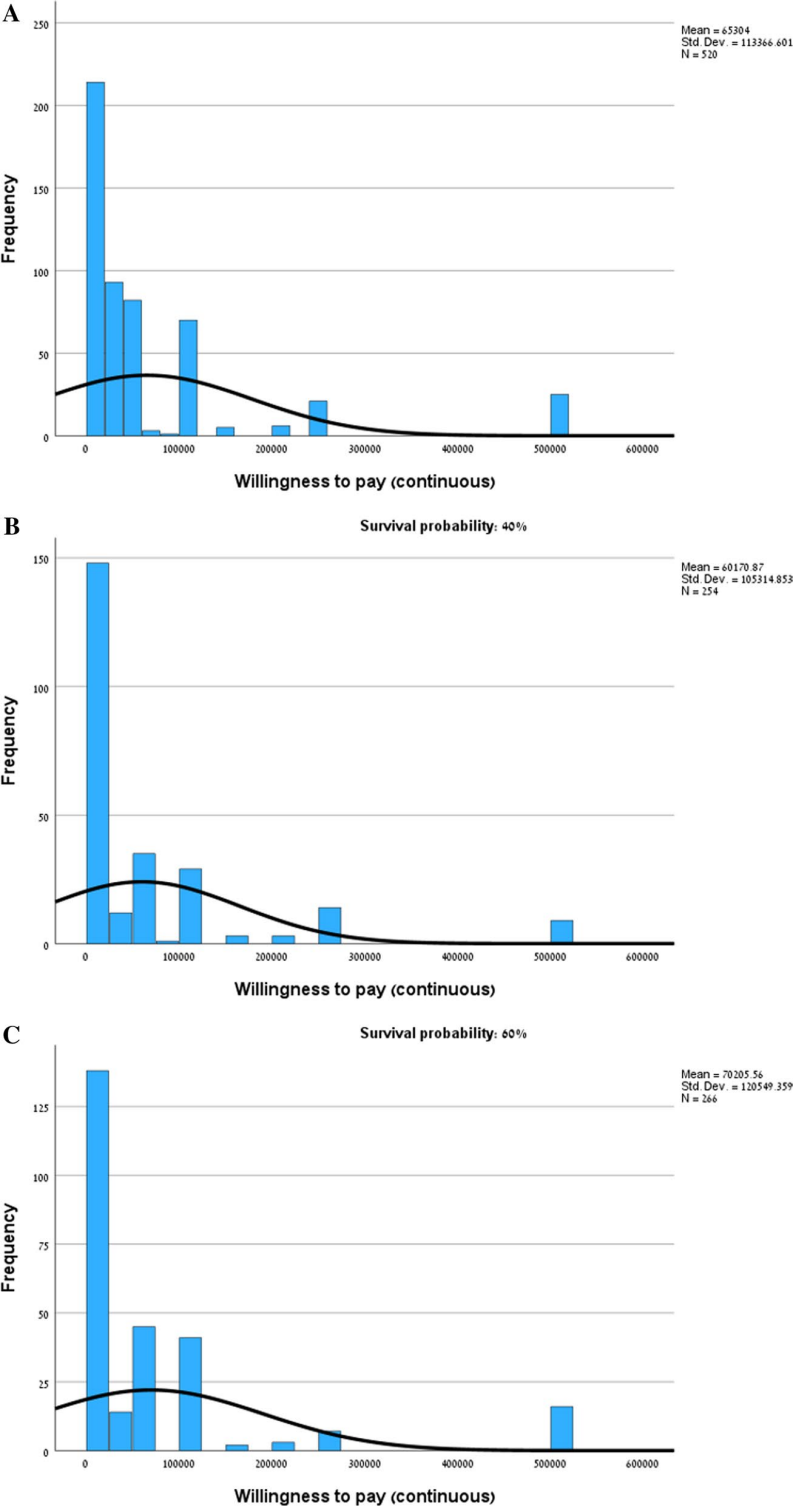
Distribution of the maximum WTP for the hypothetical mRNA treatment. **A** Entire sample, **B** Survival probability from current 20% to potential 40%, **C** Survival probability from current 20% to potential 60%

The mean, median and mode WTP for the hypothetical treatment in both scenarios (increase in survival rate from the current 20% to 40% or 60%) were ILS65,304 (± ILS113,367), ILS20,000 and ILS50,000, respectively (1USD = 3.4ILS). Minimum WTP was 0. In order to avoid inflated WTP values, the maximum WTP was trimmed at ILS 500,000, although 15 respondents were willing to pay more than the defined ceiling (Fig. 1). The WTP was skewed towards zero (skewness = 2.853), and 9.6% of respondents were not willing to pay any amount. While the median WTP in each scenario was equal to ILS20,000, the mean WTP in the 60% scenario (ILS70,206 ± ILS120,549) was somewhat higher than the mean WTP in the 40% scenario (ILS60,171 ± ILS105,315), however differences were not statistically significant. Also, mode in the 60% scenario (ILS50,000) was higher than the mode in the 40% scenario (ILS20,000). In both scenarios WTP ranged from 0 to ILS500,000.

### Characteristics associated with WTP

Table 2 presents the association between the respondents’ characteristics, stated WTP bid (continuous variable) and WTP any amount for the proposed mRNA-based anti-cancer treatment. Age was found to be significantly associated with the monetary value of the respondents’ WTP: there was a slight negative Pearson correlation of −0.159 (*p* < 0.001); i.e., younger people were willing to pay more. Higher WTP amounts were also significantly associated with higher income (*p* < 0.001), self-reports of being in good health (*p* < 0.05), having supplementary health insurance (*p* < 0.05), Jews compared to other populations (*p* < 0.01), being technology-oriented (*p* < 0.001) and tending to adopt medical innovations (*p* < 0.001).

**Table 2:** Characteristics associated with WTP (independent sample tests)

Group	Variable		Continuous WTP			Dichotomous WTP	
	variable	Baseline group	Correlation	Asymptotic Sig.(2-sided test)	Significance	Asymptotic Sig.(2-sided test)	Significance
Demographics	Age (continuous)		− 0.159**	0.000283964	*	0.827600534	
	Age (groups)	51–34		0.188791717		0.543239782	
	Sex	Male		0.333111573		0.358983071	
	Kids	None		0.541079271		0.219903938	
	Religion	Jew		0.001961157	**	0.000102976	*
	Religiocity	Secular		0.295683067		0.113728524	
	Income	High		0.000206348	*	0.001737942	**
	Education	Academic		0.644694057		0.122174856	
Contingent	Health status	Good		0.011310297	***	0.042264041	***
	Health insurance	Basic +		0.043221254	***	0.000999077	*
	Cancer occurrence	Yes		0.147470186		0.053821657	
	Cancer type mentioned	No, but other cancer		0.418434382		0.078391950	
	Covid vaccination	Yes		0.194654014		0.002670096	**
	Heard about mRNA	Yes		0.425924988		0.011172878	***
	Survival probability	60%		0.113292428		0.097600454	
Health	Balanced nutrition	Yes		0.265603967		0.530743299	
	Exercise	Yes		0.140479396		0.840145602	
	Medical checks	No		0.830881672		0.396679991	
	Smoking	No		0.488243685		0.029353168	***
Technology	Technology attitude	Yes		0.000012049	*	0.000000771	*
	Medical innovation attitude	Yes		0.000000002	*	0.000000000	*

*p < 0.001; **p < 0.01; ***p < 0.05

Willingness to pay any amount for the proposed mRNA-based anti-cancer treatment was significantly associated with higher income (*p* < 0.01), self-reporting good health (*p* < 0.05), having supplementary health insurance (*p* < 0.001), being vaccinated for COVID-19(*p* < 0.01), familiarity with mRNA technology (*p* < 0.05), being a non-smoker (*p* < 0.05), positive attitudes toward technology (*p* < 0.001), Jews compared to other populations (*p* < 0.001), and a tendency to adopt medical innovations (*p* < 0.001). No statistical difference between the two scenarios (increased survival rate from the current 20% to 40% vs. 60%) was found either for the WTP amounts or for willingness to pay any amount for the proposed treatment.

### Regression models for the associations between respondents’ characteristics and WTP

Table 3 presents the regression models assessing the characteristics impacting respondents’ monetary WTP (continuous variable) and their willingness to pay any amount (dichotomous variable) for the proposed treatment. WTP (yes/no) was examined using logistic regression. The strength of the regression (Nagelkerke *R* square = 0.512) implies a high level of certainty in concluding an association between the respondents' characteristics and WTP.

**Table 3:** Characteristics associated with WTP (OLS and logistic regression)

Group	Variable		OLS regression					Logistic regression				
	Variable	Baseline group	Coefficient	Significance	Significance	95.0% Confidence interval for B		Exp(B)	Significance	Significance	95% CI for EXP(B)	
						Lower bound	Upper bound				Lower	Upper
	Constant		111,689									
Demographics	Age (continuous)	–	(1,415)	0.000	*	(2,064)	(766)	0.98	0.034	***	0.97	1.00
	Religion	Jew	27,498	0.027	***	3,088	51,909	2.42	0.001	*	1.44	4.06
	Income	High	28,524	0.038	***	1,573	55,476	3.36	0.009	**	1.35	8.36
Contingent	Health insurance	Basic +			–			1.69	0.014	***	1.11	2.57
	Cancer type mentioned	No, but other cancer			–			1.71	0.050		1.00	2.92
	Heard about mRNA	Yes	(17,077)	0.093		(37,040)	2,887			–		
	Survival probability	0.6			–			1.38	0.092		0.95	2.01
Health	Medical checks	No	21,648	0.088		(3,205)	46,500			–		
	Smoking	No			–			0.45	0.034	***	0.21	0.94
Technology	Technology attitude	Yes	(31,107)	0.083		(66,243)	4,029	0.47	0.037	***	0.23	0.95
	Medical innovation attitude	Yes	(33,664)	0.012	***	(59,900)	(7,429)	0.16	0.000	*	0.07	0.35

*p < 0.001; **p < 0.01; ***p < 0.05

The existence of WTP (any amount) was significantly associated with younger vs. older (*p* < 0.05), Jews vs. other populations (*p* < 0.001), higher income (*p* < 0.01), having supplementary health insurance (*p* < 0.05), being a non-smoker (*p* < 0.05), positive attitude toward technology (*p* < 0.05) and the tendency to adopt medical innovations (*p* < 0.001).

No strong correlations (> ± 0.5) were found between variables, except for a negative correlation of −0.520 between age and having supplementary health insurance. The regression strength was quite low (adjusted *R* square = 0.065), due to the WTP distribution which deviated from a normal distribution because of the skewedness towards zero. Quantile regression was run as an alternative model; however, in this case as well, the model fit was relatively low: Pseudo *R* squared values: Q = 0.1–0.009; Q = 0.25–0.036; Q = 0.5–0.040; Q = 0.75–0.069; Q = 0.9–0.154.

Higher monetary WTP was significantly associated with higher income (*p* < 0.05), younger vs. older (*p* < 0.001), Jews vs. other populations (*p* < 0.05), and the tendency to adopt medical innovations (*p* < 0.05).

## Discussion

The main rationale for this WTP study derives from the surge of interest in mRNA-based interventions generated by the success of COVID-19 vaccines. The influence of mRNA-based therapy is expected to grow in the coming decades and provide new treatment options for a variety of indications, including cancer. Despite the scientific breakthroughs in cancer treatment in the last 20 years, many types of cancer are still incurable. Recently, an investigational personalized mRNA cancer vaccine combined with Keytruda, Merck's anti-PD-1 therapy was granted Breakthrough Therapy Designation by the U.S. Food and Drug Administration (FDA) for the adjuvant treatment of patients with high-risk melanoma following complete resection [34]. The FDA's Breakthrough Therapy Designation is designed to expedite the development and review of drugs that are intended to treat a serious condition, and when preliminary clinical evidence indicates that the product may demonstrate substantial improvement over available therapies on at least one clinically significant endpoint [35].

There is clearly a need to examine the public’s perception of potential innovative interventions since patient and public involvement can support and enhance healthcare decision-making at the individual, policy, and commissioning levels. In particular, WTP research can provide payers and regulators with evidence-based tools to inform policymaking, including future coverage and reimbursement decisions. Innovation is often invoked by the pharmaceutical industry as an argument to justify higher prices for new products. While innovation is a very broad notion, one of its most important dimensions is the added health benefits of new treatments. The value of these added benefits is a key component in pricing and by extension the amount of money society or individuals are willing to pay for it. This study assesses broadly the WTP for a therapy with a likelihood cure and has a wider implication beyond the specific case of mRNA technology.

Nevertheless, individual factors and considerations are likely to affect WTP for mRNA-based anti-cancer treatment. Cancer patients, for example, vary in their risk appetite. While some ("type 1") value a treatment characterized by a wide range of potential outcomes (hoping they will be lucky), others ("type 2") prefer treatment with more certainty. For instance, "type 1" patients may be willing to pay a higher price for a novel immuno-oncology treatment that will provide durable survival for a minority of the population. By contrast, "type 2" patients may be willing to pay more for a standard of care with known outcomes: a few months of survival for the majority of patients. This concept was tested in a population-based survey in the U.S. comparing WTP for an increase in median survival time to an increase in the probability of survival for a given length of time (landmark survival) [36]. The results indicated that respondents’ WTP was substantially higher for landmark survival when a cancer-related scenario was presented. In another study, Lakdawalla and colleagues [37] found that cancer patients preferred treatment with a small chance of a large survival gain over treatments with a similar average survival rate but a smaller chance of a large gain. Patients at the end of their lives switch from being risk-averse to risk-lovers and are more willing to gamble.

Another important consideration is the “option value”. Patients may find value in treatments that extend their survival prospects until another effective treatment is available. This concept of “buying time” makes sense for life-threatening diseases with fast-growing clinical research and development, such as certain types of cancer. “Real option value” is generated when a health technology that extends life creates opportunities for the patient to benefit from future advances in medicine [38]. This was found to be the case for metastatic melanoma patients, when the utilization of existing treatments was modified after disclosure of the then-investigational drug Ipilimumab's clinical trial results [39].

Several related WTP studies have been conducted in Israel. In response to a public debate in 2014, the Israel Cancer Association conducted a survey examining the willingness of patients to increase their monthly supplementary health insurance premium to finance non-reimbursed life-saving medications. The results showed [40] that about half of the population was willing to pay ILS50 per month for this purpose, with variations depending on patients' age and socio-economic status. A previous survey showed that Israeli adults were willing to pay extra health fees (up to 7.5% of the average health fee) to guarantee that all life-saving and life-extending interventions would be included in the National List of Health Services (NLHS) at no copayment [41].

To the best of our knowledge, this is the first study in Israel exploring WTP for a potential innovative technology to treat cancer. The results suggest that the public is willing to pay more for better performing anti-cancer therapies. Significant added benefits are seen as a sufficient rationale for higher levels of WTP, reimbursement and coverage recommendations. The mean out-of-pocket WTP in the 60% scenario was somewhat higher (ILS70,206 ± ILS120,549) than the current to 40% scenario (ILS60,171 ± ILS105,315), while differences were not statistically significant. The WTP of the population examined in the current study for a hypothetical innovative mRNA-based treatment was primarily associated with higher income, good health, familiarity with the technology and tendency to adopt innovative interventions. The absence of multicollinearity between variables suggests that the model accounting for WTP is likely to be robust and valid.

This association between WTP for cancer-related interventions and demographic characteristics is consistent with other studies. A number of studies have indicated that WTP monetary values are positively influenced by race/ethnicity [42–46], similar to our findings for the WTP of Jews versus other ethnic groups. As found here, most studies have shown that WTP amounts increase with respondent income [17, 31, 35, 44, 46–81].

### Limitations

This study has a number of limitations. There was a slightly higher representation of young, secular and educated strata in our sample compared to the general Israeli population. Also, the proportion of Muslims in our study sample (10%) is lower compared with their representation in the Israeli population, thus Muslims are underrepresented in our study. This is typical of web-based panels where the participants tend to be relatively more technology-oriented [82, 83]. Nevertheless, the overall socio-demographic distribution of our respondents seemed reliable, and the level of representativeness of the Israeli public was acceptable. The second limitation concerns the fact that this study relied on the participants’ self-reported mRNA familiarity and attitudes toward technology. This kind of potential bias has received a great deal of attention in behavioral and medical studies. People may provide inaccurate assessments of their own knowledge for a variety of reasons, from simple misinterpretation to social-desirability bias [84]. Despite this concern, the success of the COVID-19 mRNA-based vaccination in Israel and the reputation of Israeli society as a “start-up” nation suggest that this risk was relatively low. Third, in the case of contingent valuation studies, difficulties in understanding future hypothetical medical interventions might undermine respondents’ ability to accurately estimate their WTP [85]. However, this potential problem impacts all other WTP elicitation techniques as well. This is why in general, decisions relying on WTP results should not only consider the statistical significance of these outcomes but also their policy context and intended use. Nonetheless, WTP is being used more and more frequently in real-world settings, thus confirming the reliability and validity of this methodology. Finally, the generalization of the findings to other countries may be curtailed by the differences in Israel's demographic characteristics. Thus, since mRNA-based health technologies are entering the market in many countries, more WTP studies should be conducted at the national level to inform local stakeholders. Lastly, due to WTP skewness towards zero it is unable to solely rely on mean WTP values but rather requires looking at complementary statistical measures such as median, mode and range. Notwithstanding these limitations, the results of this study suggest that Israeli citizens are willing to pay relatively high amounts for access to a new intervention for cancer treatment that is not yet on the market. Similar studies should be conducted in other countries with different healthcare systems and population characteristics.

## Conclusion

Israel is known to be an early adopter of novel technologies and was recently a global pioneer in its massive COVID-19 vaccination campaign using mRNA technology. Prior to 2021, mRNA was perceived as an immature technology, but after the emergency authorization by the FDA and EMA, it was widely implemented at the fastest pace in the history of medicine. Today, this technology is eliciting intense interest and there are ongoing accelerated clinical trials examining its applicability for other therapeutic areas. The outcomes of the cancer treatment survey proposed here can thus be harnessed by policymakers, the pharmaceutical industry and other stakeholders. Given the expected surge in innovative treatment based on the mRNA technology, applications for coverage and reimbursement of these interventions are likely to arise in the coming years. Currently, a range of considerations including, among others, the strength of supporting evidence on treatment efficacy, unmet medical need, disease severity, as well as cost and value for money considerations are incorporated in coverage and reimbursement decisions. However, to date, patients’ and societal preferences including WTP assessments, have been given only limited weight in these decisions, both in Israel and in other countries. We believe that assessing and incorporating societal views and preferences will broaden the scope of considerations used in coverage and reimbursement committees, aligning decisions more closely with the values and priorities of the population it serves, allowing more equitable resource allocation decisions. In this context, our research is the very first to explore societal views on WTP for mRNA-based anti-cancer treatments. Similar studies conducted in other healthcare systems could foster early dialogue and collaboration at the global level about patient access to and payment models for upcoming mRNA-based interventions.

## Data Availability

The datasets used and/or analyzed during the current study are available from the corresponding author on reasonable request.

## Appendix 1: Questionnaire

### WTP for mRNA anti-cancer treatment questionnaire

**A. Risk tolerance**

1. Do you eat a balanced, healthy diet?
  1. Always
  2. Usually
  3. Sometimes
  4. Seldom
  5. Never

2. Do you get physical exercise?
  1. Frequently
  2. Usually
  3. Sometimes
  4. Seldom
  5. Never

3. Do you get periodic medical tests?
  1. Always
  2. Usually
  3. Sometimes
  4. Seldom
  5. Never

4. Do you smoke?
  1. One pack a day or more
  2. Up to one pack a week
  3. Occasionally
  4. Seldom
  5. Never

**B. Attitude towards new technologies**

5. Generally, what is your attitude towards innovative technologies?
  1. Early adopter of new technologies
  2. I tend to adopt new technologies
  3. I adopt a new technology once it has been validated
  4. Refrain from using new technologies, I only use them once they become commonplace
  5. I don’t use new technologies

6. What is your attitude towards innovative medical treatment?
  1. Willing to try an innovative treatment immediately
  2. Willing to try innovative treatments only after a period of time and when they are reimbursed
  3. Only willing to use standard medical care
  4. Try not to use any kind of medical treatment

**C. Contingent Valuation Scenarios**

7. Generally, how would you rate your health?
  1. Excellent
  2. Very good
  3. Good
  4. Not very good
  5. Poor

8. Which kind(s) of health insurance do you have?
  6) Basic health fund insurance alone
  7) Complementary health fund insurance
  8) Collective private insurance (from the workplace)
  9) Personal private insurance (out of my own pocket)

9. Have you or one of your relatives or your close friends had cancer?
  1. Myself
  2. My relatives or close friends
  3. Neither myself nor my relative or close friends
  4. Prefer not to state
  5. If you responded 1 or 2, skip to question 4
  6. If you responded 3 or 4, skip to question 5

10. What type of cancer?
  1. Melanoma or another type of skin cancer
  2. Lung cancer
  3. Breast or ovarian cancer
  4. Cancer of the digestive system
  5. Other tumor
  6. Blood cancer (Leukemia, Myeloma, Lymphoma)

11. Have you been vaccinated for Covid-19?
  1. I received 1 dose
  2. I received 2 doses
  3. I received 3 or 4 doses (including the booster)
  4. I haven't been vaccinated
  5. If you responded 1/2/3, skip to question 7
  6. If you responded 4, skip to question 6

12. Why haven't you been vaccinated?
  1. I had Covid-19
  2. Health constraints
  3. I don't believe in vaccinations
  4. Ideological convictions
  5. No real reason
  6. Prefer not to state

13. Have you heard about mRNA (messenger RNA) technology?
  1. I've heard about it; I am very familiar with it
  2. I've heard about it; I know a little about it
  3. I haven't heard about it.

### mRNA Scenario Background

mRNA technology is one of the outcomes of research on the human genome. Prior to the COVID-19 pandemic, mRNA had little practical medical use. At the end of 2020, the leading health authorities in the U.S. (FDA) and Europe (EMA) approved the commercialization of Pfizer’s and Moderna’s Covid-19 vaccine based on this technology. These are the vaccines used in Israel.

### Scenario

Now imagine the following theoretical scenario: you contact your general practitioner with complaints about pain and fatigue. After a series of tests, the GP tells you that you have advanced melanoma (skin cancer), lung cancer, breast/ovarian cancer or cancer of the digestive system. In terms of treatment options, the physician suggests either immunotherapy (medication that boosts the body's natural immune system to fight the tumor), or a combination of immunotherapy and mRNA technology. The first, which is fully reimbursed by your health fund, gives you a 20% chance of 5-year survival. The second involves the addition of mRNA treatment, which is not covered by the basic health insurance package, but has been approved by health regulatory authorities in the US (FDA) and in Europe (EMA). It is expected to lead to a 40% (for half of the respondents)/60% (for the second half of respondents) 5-year survival rate.

Here is a graphic illustrating the probability of a cure (5-year survival) of the new treatment versus current treatment:

**Current treatment**

**Suggested treatment (for half of the respondents)**

**Suggested treatment (for the other half of the respondents)**

Both treatments take place in the hospital on a monthly basis and have a similar adverse events profile. If you are interested in the second option, you will have to pay for it out of your own pocket.

Would you agree to pay XXX (*) annually for the new treatment, which is not covered by your health insurance?

4) Yes
5) No, but I would be willing to pay a lower amount
6) No, I would not be willing to pay any amount

14. (*) XXX –50 respondents from each group (~ 250 respondents per group of 40%/60% probability) read a text with the following amounts: NIS20,000; NIS50,000; NIS100,000; NIS250,000; NIS500,000 (overall 10 subgroups).

15. Open question: what is the maximal amount you would be willing to pay for this treatment?

………………………………………………………………………………..

16. Open question: why did you choose this specific amount?

…………………………………………………………………………………

## Abbreviations

mRNA: Messenger-RNA
NSCLC: Non-small cell lung cancer
QALY: Quality-adjusted life year
WTP: Willingness to pay
CVM: Contingent valuation method
DCE: Discrete-choice experiment
OLS: Ordinary least squares
FDA: Food and Drug Administration
NLHS: National List of Health Services
